# Clinical Implications of Associations between Headache and Gastrointestinal Disorders: A Study Using the Hallym Smart Clinical Data Warehouse

**DOI:** 10.3389/fneur.2017.00526

**Published:** 2017-10-03

**Authors:** Sang-Hwa Lee, Jae-June Lee, Youngsuk Kwon, Jong-Ho Kim, Jong-Hee Sohn

**Affiliations:** ^1^Department of Neurology, Chuncheon Sacred Heart Hospital, Hallym University College of Medicine, Chuncheon-si, South Korea; ^2^Department of Anesthesiology and Pain Medicine, Chuncheon Sacred Heart Hospital, Hallym University College of Medicine, Chuncheon-si, South Korea

**Keywords:** headache, migraine, tension-type headache, gastrointestinal disorder, gastrointestinal endoscopy, *Helicobacter pylori*

## Abstract

**Background:**

The brain and gastrointestinal (GI) tract are strongly connected *via* neural, endocrine, and immune pathways. Previous studies suggest that headaches, especially migraines, may be associated with various GI disorders. However, upper GI endoscopy in migraineurs has shown a low prevalence of abnormal findings. Also, the majority of studies have not demonstrated an association between *Helicobacter pylori* (HP) infection and migraine, although a pathogenic role for HP infection in migraines has been suggested. Further knowledge concerning the relation between headaches and GI disorders is important as it may have therapeutic consequences. Thus, we sought to investigate possible associations between GI disorders and common primary headaches, such as migraines and tension-type headaches (TTH), using the Smart Clinical Data Warehouse (CDW) over a period of 10 years.

**Methods:**

We retrospectively investigated clinical data using a clinical data analytic solution called the Smart CDW from 2006 to 2016. In patients with migraines and TTH who visited a gastroenterology center, GI disorder diagnosis, upper GI endoscopy findings, and results of HP infection were collected and compared to clinical data from controls, who had health checkups without headache. The time interval between headache diagnosis and an examination at a gastroenterology center did not exceed 1 year.

**Results:**

Patients were age- and sex-matched and eligible cases were included in the migraine (*n* = 168), the TTH (*n* = 168), and the control group (*n* = 336). Among the GI disorders diagnosed by gastroenterologists, gastroesophageal reflux disorder was more prevalent in the migraine group, whereas gastric ulcers were more common in the migraine and TTH groups compared with controls (*p* < 0.0001). With regard to endoscopic findings, there were high numbers of erosive gastritis and chronic superficial gastritis cases in the migraine and TTH groups, respectively, and the severity of gastritis was significantly higher in patients with TTH compared with controls (*p* < 0.001). However, no differences were observed in the prevalence of HP infection between the groups.

**Conclusion:**

The observed association in this study may suggest that primary headache sufferers who experience migraines or TTH are more prone to GI disorders, which may have various clinical implications. Further research concerning the etiology of the association between headaches and GI disorders is warranted.

## Introduction

Cells of the brain and gut develop almost simultaneously into the central nervous system and enteric nervous system, respectively, during gestation. They remain strongly connected *via* neural, endocrine, and immune pathways. The communication between the brain and gut, known as the gut–brain axis, is bidirectional (1–3). A recent finding on the role of gut microbiota in the gut–brain axis suggests that the gut microbiome may be associated with brain functions and neurological diseases, such as migraines (4).

Previous studies have suggested that migraines may be associated with various gastrointestinal (GI) disorders, including gastroparesis, irritable bowel syndrome, peptic ulcers, and celiac disease (5–7). While nearly all observational studies have focused on migraines, the Head-HUNT study investigated the relationship between GI symptoms and headache, including migraines. All GI complaints were as common among persons with non-migraine headaches as in migraineurs, and the association between headaches and GI complaints increased with increasing headache frequency (8).

However, an extremely low prevalence of abnormal findings on upper GI endoscopy in migraineurs has been reported previously (9, 10), although a possible association between migraines and GI disorders has been reported on the basis of both epidemiological data and pathophysiological considerations. These studies have suggested that GI symptoms in migraineurs were a consequence of the migraine attack.

Moreover, published reports present inconclusive data concerning the relationship between migraines and *Helicobacter pylori* (HP) infection. Some studies have reported a strong positive correlation, whereas others have indicated negative results. Although a meta-analysis found a trend of more frequent HP infections in patients with migraines, the data are limited and investigations have focused only on subgroups of patients and patients of different ethnicities (11).

The association between GI symptoms and headache is frequently unrecognized. Further knowledge concerning headaches and GI disorders is important as it may have therapeutic consequences. Thus, we sought to investigate the possible associations between GI disorders and the common primary headache, including migraines and tension-type headaches (TTH), using the Smart Clinical Data Warehouse (CDW) over a period of 10 years.

## Materials and Methods

We retrospectively investigated clinical data using the clinical big data analytic solution Smart CDW from Hallym University Medical Center (HUMC). The smart CDW is based on the QlikView Elite Solution and is used at the six hospitals of HUMC as of 2016. It offers electronic medical record text data analysis and integrated analysis of fixed data. Thus, using the Smart CDW, we collected clinical data of patients with common primary headaches, such as migraines and TTH, and of controls who had general health checkups without headaches from January 2006 to August 2016 at the Chuncheon Sacred Heart Hospital of HUMC.

Case subjects (migraines or TTH) were eligible for inclusion if they met all of the following criteria: age 19–80 years, exclusion of a secondary cause of headache after comprehensive brain evaluation (brain computed tomography, brain magnetic resonance imaging, and/or cervical-spine X-ray), diagnosis of migraine or TTH by board-certificated neurologists at the neurology department, first visit because of headache, and ≥2 consecutive visits to the neurology department. Case subjects with a probable diagnosis or medication-overuse headache were excluded. Diagnosis of headache and its subtypes was performed by reference to the International Classification of Headache Disorders (ICHD) second or third edition (beta version) depending on the time of patient enrollment (2006–2012: ICHD II; 2013–2016: ICHD 3-beta) (12, 13). Of all enrolled subjects, we collected the medical records of those who visited the gastroenterology center. In patients with migraines and TTH who visited a gastroenterology center, GI disorder diagnosis, upper GI endoscopy findings, and results of HP infection were collected. We also selected patients who visited the gastroenterology center ≥2 consecutive times to ensure a more definite diagnosis. Thus, physician-diagnosed GI disorders included clinically presumptive or definite diagnoses after work-ups for patients who visited the gastroenterology center ≥2 times consecutively. Endoscopic diagnoses included findings specific to the esophagus, stomach, and duodenum. Infection with HP was determined using the rapid urease [*Campylobacter*-like organism (CLO)] test. The time interval between primary headache diagnosis and gastroenterology examination did not exceed 1 year. The control group included patients aged 19–80 years who had undergone general health checkups at a health promotion center, excluding patients with a history of headaches, which was assessed using a basic questionnaire completed prior to the health examination and patients with a history of headache, who visited our hospital (control group 1). We collected results of upper GI endoscopy and of the CLO test, which were performed during a general checkup. We also included subjects who visited the gastroenterology center within 1 year of a health checkup in the control group (control group 2), and collected their medical records, including diagnoses made by gastroenterologists. The GI disorder diagnoses, upper GI endoscopy findings, and results of HP infection of migraine, TTH, and control groups were collected and compared. This study design was reviewed and approved by the Institutional Review Board.

### Statistics

The R statistical software ver. 3.3.2 (R Foundation for Statistical Computing, Vienna, Austria) was used for all analyses and a value of *p* < 0.05 was considered to indicate statistical significance. The migraine and TTH group were age- and sex-matched at a 1:1 ratio and subsequently combined into one group (the headache group), which was matched to the control group. As a result, the data from the migraine, TTH, and control groups were matched in a 1:1:2 ratio. After continuous variables were converted to categorical variables using 5-year age intervals, exact matching was performed, which matched age and gender precisely. The mean and SD of continuous variables and the number and ratio of subjects of categorical variables are presented. For continuous variables, the two-sample *t*-test or Wilcoxon rank sum test were used to compare two groups, and one-way analysis of variance or the Kruskal–Wallis test were used to compare three groups. The Chi-square or Fisher’s exact tests were used for comparisons of categorical variables between groups. When differences between the three groups were significant, the Bonferroni correction was performed to test for differences in each pair.

## Results

### Subjects

A total of 2,637 patients with migraines and 6,986 patients with TTH were enrolled in this study using the CDW over a 10-year period (January 1, 2006 to August 31, 2016). In these patients, a total of 280 patients with migraines (10.6%) and 661 patients with TTH (9.4%) had medical records indicating a visit to a gastroenterology center within 1 year. Moreover, 8,524 controls without headache history who underwent a general health checkup and 1,650 control subjects who visited a gastroenterology center within 1 year of their health checkup were enrolled in control groups 1 and 2, respectively (Table 1). After age and sex matching, eligible subjects were included in the migraine (*n* = 168), TTH (*n* = 168), and control groups 1and 2 (*n* = 336, *n* = 336) (Table 2).

**Table 1:** Subjects’ characteristics in the different diagnostic groups before age and sex matching.

	Migraine (*N* = 280)		TTH (*N* = 661)		Control 1^a,b^ (*N* = 8,524)		Control 2^a,b^ (*N* = 1,690)	
Age	*N*	%	*N*	%	*N*	%	*N*	%
≤39	107	38.21	112	16.94	9	0.11	274	16.21
40–44	39	13.93	58	8.77	470	5.51	180	10.65
45–49	44	15.71	78	11.80	1,088	12.76	173	10.24
50–54	38	13.57	101	15.28	1,106	12.98	306	18.11
55–59	22	7.86	76	11.50	1,649	19.35	265	15.68
60–64	7	2.50	65	9.83	1,463	17.16	172	10.18
65–69	12	4.29	71	10.74	914	10.72	136	8.05
70–74	6	2.14	47	7.11	929	10.90	136	8.05
≥75	5	1.79	53	8.02	896	10.51	48	2.84

Mean (SD)	43.49 (13.59)		53.96 (14.23)		60.15 (10.49)		52.38 (12.98)	

**Sex**	*****N*****	**%**	*****N*****	**%**	*****N*****	**%**	*****N*****	**%**

Male	54	19.29	251	37.97	3,769	44.22	907	53.67
Female	226	80.71	410	62.03	4,755	55.78	783	46.33

*^a^Significant difference between the headache group and the control groups 1, 2 (*p* < 0.0001)*.

*^b^Significant difference between patients with migraines and TTH and the control groups 1, 2 (*p* < 0.0001)*.

*TTH, tension-type headache*.

**Table 2:** Subject characteristics in the different diagnostic groups after age and sex matching.

	Migraine (*N* = 168)		TTH (*N* = 168)		Control 1^a,b^ (*N* = 336)		Control 2^a,b^ (*N* = 336)	
Age	*N*	%	*N*	%	*N*	%	*N*	%
≤39	3	1.79	3	1.79	6	1.79	6	1.79
40–44	32	19.05	32	19.05	64	19.05	64	19.05
45–49	44	26.19	44	26.19	88	26.19	88	26.19
50–54	38	22.62	38	22.62	76	22.62	76	22.62
55–59	22	13.10	22	13.10	44	13.10	44	13.10
60–64	7	4.17	7	4.17	14	4.17	14	4.17
65–69	12	7.14	12	7.14	24	7.14	24	7.14
70–74	6	3.57	6	3.57	12	3.57	12	3.57
≥75	4	2.38	4	2.38	8	2.38	8	2.38

Mean (SD)	51.83 (9.79)		51.75 (9.40)		52.36 (9.25)		51.95 (9.35)	

**Sex**	*****N*****	**%**	*****N*****	**%**	*****N*****	**%**	*****N*****	**%**

Male	27	16.07	27	16.07	54	16.07	54	16.07
Female	141	83.93	141	83.93	282	83.93	282	83.93

*^a^No significant difference between the headache group and the control groups 1, 2*.

*^b^No significant difference between patients with migraines and TTH and the control groups 1, 2*.

*TTH, tension-type headache*.

### Comparison of GI Disorders in the Headache and Control Groups

The GI disorder diagnoses made by gastroenterologists in the headache and control groups were compared. Among the GI disorders, gastroesophageal reflux disorder (GERD) was more prevalent in the migraine group, whereas gastric ulcers were more common in the migraine and TTH groups compared with the control group (*p* < 0.0001) (Table 3). When headache patients were grouped according to migraine and TTH subtypes (migraine with aura, migraine without aura, or chronic migraine; episodic or chronic TTH), significant differences were found between the groups in terms of GI disorder diagnoses (*p* = 0.0085). Multiple comparisons using the Bonferroni correction showed only significant differences between the migraine without aura and episodic TTH groups (*p* = 0.0418) (Table 4).

**Table 3:** Comparison of physician-diagnosed GI disorders between the headache groups and controls.

GI disorder diagnosis	Migraine^a,c^ (***N*** = 168)		TTH^b,c^ (*N* = 168)		Control^a,b^ (*N* = 336)		*p*-Value^1^	*p*-Value^2^
	*N*	%	*N*	%	*N*	%		
GERD	45	26.79	20	11.90	36	10.71	<0.0001	<0.0001
Gastritis	85	50.60	103	61.31	207	61.61		
Gastric ulcer	20	11.90	20	11.90	19	5.65		
IBS	3	1.79	4	2.38	2	0.60		
FGID	6	3.57	17	10.12	25	7.44		
Liver disease	0	0.00	0	0.00	21	6.25		
Etc.	9	5.36	4	2.38	26	7.74		

*p-Value^1^, headache group vs. control group*.

*p-Value^2^, migraine group vs. TTH groups vs. control group*.

*GI, gastrointestinal; TTH, tension-type headache; GERD, gastroesophageal reflux disorder; IBS, irritable bowel syndrome; FGID, functional gastrointestinal disorder*.

*^a^Significant differences between the migraine group and controls by multiple comparisons using Bonferroni correction *(p* < 0.0001)*.

*^b^Significant differences between the TTH group and control group by multiple comparisons using Bonferroni correction (*p* = 0.0008)*.

*^c^Significant differences between the migraine and TTH groups by multiple comparisons using Bonferroni correction (*p* = 0.0067)*.

**Table 4:** Comparison of physician-diagnosed GI disorders in patients with migraine and TTH subtypes.

Diagnosis of GI disorder	MWA (*N* = 19)		MOA^a^ (*N* = 132)		CM (*N* = 17)		ETTH^a^ (*N* = 155)		CTTH (*N* = 13)		*p*-Value
	*N*	%	*N*	%	*N*	%	*N*	%	*N*	%	
GERD	3	15.79	36	27.27	6	35.29	17	10.97	3	23.08	0.0085
Gastritis	9	47.37	68	51.52	8	47.06	98	63.23	5	38.46	
Gastric ulcer	5	26.32	13	9.85	2	11.76	16	10.32	4	30.77	
IBS	0	0.00	3	2.27	0	0.00	3	1.94	1	7.69	
FGID	0	0.00	6	4.55	0	0.00	17	10.97	0	0.00	
Etc.	2	10.53	6	4.55	1	5.88	4	2.58	0	0.00	

*GI, gastrointestinal; TTH, tension-type headache; MWA, migraine with aura; MOA, migraine without aura; CM, chronic migraine; ETTH, episodic TTH; CTTH, chronic TTH; GERD, gastroesophageal reflux disorder; IBS, irritable bowel syndrome; FGID, functional gastrointestinal disorder*.

*^a^Significant differences between the migraine without aura and episodic TTH groups by multiple comparisons using Bonferroni correction (*p* = 0.0418)*.

### Comparison of Upper GI Endoscopic Findings in the Headache and Control Groups

Endoscopic diagnoses are listed in Table 5. The endoscopic findings of the esophagus and duodenum in the headache group did not differ from those of controls. However, significant differences were noted in gastric endoscopy findings between the headache and control groups in addition to those found among patients with migraines, TTH, and controls. Also, significant differences by multiple comparisons using the Bonferroni correction were seen between the headache groups and the control group (migraine vs. control *p* = 0.0071; TTH vs. control *p* = 0.0007). On gastric endoscopy, a high number of chronic superficial gastritis (CSG) cases were found in the TTH group, whereas erosive gastritis was more prevalent in patients with migraines compared with controls. Also, gastric ulcers were more prevalent in the headache group compared with the control group. Significant differences were found in the severity of gastritis between the headache groups and controls and among patients with migraines, TTH, and controls. The degree of gastritis was notably more severe in patients with TTH compared with controls, as assessed using the Bonferroni correction (*p* < 0.001). There were no differences in severity of reflux esophagitis using the Los Angeles classification between the groups (Table 5). Moreover, there were no observed differences in upper GI endoscopy findings between migraine and TTH subtypes.

**Table 5:** Comparison of upper GI endoscopic findings in the headache groups and controls.

	Migraine (*N* = 168)		TTH (*N* = 168)		Control (*N* = 336)		*p*-Value^1^	*p*-Value^2^
	*N*	%	*N*	%	*N*	%		
**Esophagus**							0.43530	0.7099
Normal	57	33.93	63	37.50	136	40.48		
Reflux esophagitis	103	61.31	98	58.33	185	55.06		
Miscellaneous	8	4.76	7	4.17	15	4.46		
**Stomach^a,b^**							<0.0001	0.0003
Normal	1	0.60	0	0.00	3	0.89		
CSG	126	75.00	137	81.55	246	73.21		
Erosive gastritis	29	17.26	20	11.90	46	13.69		
Ulcers	10	5.95	10	5.95	11	3.27		
Miscellaneous	2	1.19	1	0.60	30	8.93		
**Duodenum**							0.7232	0.7059
Normal	142	84.52	150	89.29	283	84.23		
Duodenitis	5	2.98	2	1.19	7	2.08		
Ulcer	20	11.90	14	8.33	41	12.20		
Miscellaneous	1	0.60	2	1.19	5	1.49		
**Reflux esophagitis severity (Los Angeles classification)**							0.2650	0.4851
LA-M	84	81.55	77	78.57	157	84.86		
LA-A	14	13.59	15	15.31	23	12.43		
LA-B	4	3.88	6	6.12	4	2.16		
LA-C	0	0.00	0	0.00	1	0.54		
LA-D	1	0.97	0	0.00	0	0.00		
**Gastritis severity^c^**							0.0003	0.0002
Mild	69	54.76	55	40.15	160	65.04		
Moderate	52	41.27	74	54.01	78	31.71		
Severe	5	3.97	8	5.84	8	3.25		

*p-Value^1^, headache group vs. control group*.

*p-Value^2^, migraine group vs. TTH groups vs. control group*.

*GI, gastrointestinal; TTH, tension-type headache; CSG, chronic superficial gastritis*.

*^a^Significant differences between the migraine and control groups by multiple comparisons using Bonferroni correction (*p* = 0.0071)*.

*^b^Significant differences between the TTH group and controls by multiple comparisons using Bonferroni correction (*p* = 0.0007)*.

*^c^Significant differences between the TTH and control groups by multiple comparisons using Bonferroni correction (*p* < 0.0001)*.

### Comparison of HP Infections in the Headache and Control Groups

The prevalence of HP infections was higher in patients with migraines than in the TTH and control groups, but not significantly so, and no other differences were observed in the presence of HP infections between the groups (Table 6).

**Table 6:** Comparisons of the HP positive rates between the headache groups and the controls.

	Migraine		TTH		Control		*p*-Value^1^	*p*-Value^2^
	*N*	%	*N*	%	*N*	%		
HP infection							0.5172	0.3271
Positive	51	64.56	43	54.43	89	55.28		
Negative	28	35.44	36	45.57	72	44.72		

*p-Value^1^, headache group vs. control group*.

*p-value^2^, migraine group vs. TTH groups vs. control group*.

*HP, Helicobacter pylori; TTH, tension-type headache*.

## Discussion

This study aimed to examine the association between GI disorders and common primary headaches. We enrolled eligible patients with medical records indicating gastroenterology center visits, who had experienced migraines or TTH as diagnosed by a neurologist using the Smart CDW during a period of 10 years. Among the GI disorders diagnosed by gastroenterologists, GERD was more prevalent in the migraine group, whereas gastric ulcers were more common in the migraine and TTH groups. On upper GI endoscopy, higher numbers of chronic superficial and erosive gastritis cases were found in the TTH group and migraine groups, respectively, compared with controls. Also, endoscopic findings of gastric ulcers were more prevalent in the migraine and TTH groups compared with controls. The severity of gastritis was significantly higher in patients with TTH compared with controls. However, no differences were observed in the prevalence of HP infection between the groups.

Migraines are associated with GI disorders, such as gastroparesis, GERD, irritable bowel syndrome, and celiac disease (5, 6, 14), and are also often accompanied by various GI symptoms, such as diarrhea, constipation, or dyspepsia (7, 8). In a previous large population-based study, all of the GI symptoms investigated (nausea, reflux symptoms, diarrhea, and constipation) seemed to be approximately as common among non-migraineurs as among migraine sufferers, suggesting that headache sufferers are generally predisposed to GI complaints (8). In our study, the frequency of GERD was approximately 27% in the migraine group and 10% in the control group. Gastric ulcers were much more common in the migraine and TTH groups than in the control group (migraine 11.90% vs. TTH 11.90% vs. control 5.65%). A previous web-based survey of migraine patients reported a GERD frequency of 22%, which is similar to that of our study (14). Another clinic-based study reported that the frequency of GERD was higher in migraineurs in comparison with non-migraineurs (42 vs. 18%), while gastritis and gastric ulcer prevalence were not significantly different between migraineurs and non-migraineurs (6 vs. 5.7%), which is similar to the results of our study (15). The prevalence of GERD is reported to range from 13.2 to 20% in the US, while it is 3.5% in the Korean population, which is lower than in Western countries (16, 17). Considering these data, there is a higher prevalence of GERD in migraineurs in our study. The mechanism by which GERD is increased among individuals who experience migraines is not clear, but is plausibly related to the common pathophysiological mechanisms underlying both disorders. Autonomic nervous system (ANS) dysfunction has previously been linked to both headaches, especially migraines, and GI disorders (18–23). Thus, ANS dysfunction is involved in the pathogenesis of both of these disorders, which may explain the overlap of GERD and migraines. Abnormal visceral mechanosensory and vagal function has been found to be involved in dyspeptic patients, and disturbances in visceral nerves have also been implicated in migraines (7, 24). In addition, individuals with headaches and GI symptoms had more severe and frequent symptoms compared to those subjects without GI symptoms (8, 25, 26). A possible explanation for this effect may be a heightened common pathophysiological mechanism, which may lead to more severe symptoms in individuals with dual pathology compared to just headaches alone (25).

Upper GI endoscopy revealed a high prevalence of erosive gastritis in the migraine group compared with the control group. In a previous study, completely normal endoscopic findings were found in 90% of migraineurs with nausea, vomiting, or other dysmotility-like symptoms (10). Also, a low prevalence of abnormal findings on upper GI endoscopy and esophageal PH monitoring in migraineurs has been reported. The study revealed normal findings in 45% of migraineurs and erosive gastritis in 18% of migraineurs, which is similar to our results (17.26%) (9). Erosive gastritis diagnosed by endoscopy is caused by damage to the gastric mucosa with a depressed area due to erosion on the hemispheric nodule. Although the pathophysiology of erosive gastritis has yet to be clarified, it is thought to occur due to an imbalance between the offensive factors in the stomach and the defensive factors of the stomach wall (27). To explain the association between erosive gastritis and migraines, the following mechanisms can be considered. The first is the possibility of an increased likelihood of oral medication intake, such as non-steroidal anti-inflammatory drugs (NSAIDs), as an offensive factor in migraineurs. The second possibility is that the gastric mucosa is damaged due to a delayed gastric emptying time associated with gastroparesis in migraineurs. A previous report found that delayed gastric emptying/gastroparesis itself plays a significant role in the evolution of GERD (28). Studies have also demonstrated that gastric stasis is present among migraineurs both during and outside of migraine attacks, which is suggestive of underlying GERD (14, 29). Thus, the high prevalence of pre-existing GERD in migraineurs could be associated with an increased risk of endoscopic erosive gastritis.

In a previous study, endoscopic findings were classified as normal, consistent with peptic lesions (reflux esophagitis, gastric ulcer, or duodenal ulcer), positive for polyps and/or tumors, and miscellaneous (10). Thus, if our classification, including CSG, were to be categorized as normal findings, the proportion of normal endoscopic results would be similar to that of previous studies. However, in our study, the frequency of CSG was significantly higher in the TTH group, and the severity of gastritis was significantly higher in patients with TTH compared with the control group. Also, similar to physician-diagnosed GI disorders, endoscopic findings of gastric ulcer were slightly more common in migraine and TTH patients. Although there is little evidence demonstrating an association between headache and gastritis-gastric ulcer, peptic ulcer diseases, including gastric and duodenal ulcers were previously more frequently observed in headache patients than controls (30). Gastritis-gastric ulcer in headache patients may represent side effects caused by medications taken to treat headaches, such as NSAIDs. Moreover, psychological factors may link headaches and gastritis-gastric ulcers, as both are associated with anxiety and mental disorders (31–34).

In addition, we investigated the association between headaches and HP infection. HP infection is strongly associated with gastric cancer and gastric and duodenal ulceration (35). The association between HP infection and various extraintestinal pathologies, such as coronary heart disease, migraines, Alzheimer’s disease, and mild cognitive impairment, has been addressed, but results remain controversial (36). A previous preliminary study reported that 40% of patients diagnosed with primary headache were seropositive for HP (37). However, the majority of investigations have focused on the relationship between HP infection and migraines. A previous meta-analysis of five case–control studies showed that the prevalence of HP infection was significantly greater in patients with migraines than in controls (44.97 vs. 33.26%). Also, several studies have suggested that HP eradication treatment may provide migraine headache relief (38–40). Stratification analysis by geographical region has previously indicated that HP infection levels among migraineurs were greater in Asia (11). Similar to previous research, the prevalence of HP infection was greater in migraineurs in our study compared with European studies (35, 39, 41). However, a relatively higher prevalence was found in the TTH and control groups in our study compared to previous studies (35, 36, 39–41). This phenomenon might result from a significantly greater prevalence of HP cagA-positive strains in Asian countries (42). In a previous study, the prevalence of HP infection assessed using the 13C-urea breath test, which is used in clinical practice, was similar in migraine patients and in controls, while HP cagA-positive strains detected through enzyme-linked immunosorbent assays were strongly associated with migraines with aura (39). Although epidemiologic evidence suggests an association between migraines and HP infection, investigations have only focused on patient subgroups and those of different ethnicities. Indeed, the data are limited (11, 43). Thus, it is warranted to consider the regional variations in HP infection, and further studies including larger sample sizes are needed.

Some studies have reported an association between primary headaches and GI disorders, and have also demonstrated remission or improvement of headaches following treatment of the accompanying GI disorders (38, 44–47). However, the scientific literature investigating mechanisms underlying the comorbidity of the two conditions is scant. Hypotheses explaining this association implicate central sensitization and parasympathetic referred pain, serotonin pathways, ANS dysfunction, systemic vasculopathy, and food allergies (48). In a previous study investigating associations between headache presence and cellular changes in the gastroduodenal mucosa, the presence of headache associated with dyspeptic symptoms was strongly related to mucosal mast cell density in pediatric patients with HP-negative functional dyspepsia. Thus, this observed association suggests a functional link between the brain and the GI tract (49). Further epidemiologic, clinical, and pathophysiologic evidence is needed to reveal associations between headaches and GI disorders.

This study has several limitations. First, it used data collected from individuals who visited a single university hospital in a regional community. Therefore, it is difficult to generalize the study results to the general population, and the possibility of selection bias must be considered. Next, the mean age of the migraine group was higher than that of similar previous studies due to age matching with the TTH group. Finally, our study design was retrospective, and, unfortunately, we did not collect exact clinical information regarding headache characteristics, such as frequency, duration, or medication history (NSAIDs or analgesics). Future prospective, population-based studies are needed to further investigate the association between headache and GI disorders.

In conclusion, the association observed in our study may suggest that common primary headache, including migraine and TTH, sufferers are more prone to GI disorders. Specifically, GERD and endoscopic erosive gastritis were more frequent in individuals with migraines, whereas endoscopic CSG was more frequent in the TTH group, and was associated with more severe gastritis. In addition, gastric ulcers were more prevalent in the migraine and TTH groups than the control group. Comorbid GI disorders in headache patients may have clinical implications and may also affect therapeutic outcome. Thus, it is important to consider accompanying GI disorders and symptoms in patients with headaches.

## Ethics Statement

The protocol was approved by the Hallym University Chuncheon Sacred Heart Hospital IRB.

## Author Contributions

Conception and design of the work: J-HS and S-HL. Acquisition of data for the work: J-JL and J-HK. Analysis and interpretation of data for the work: J-HS, S-HL, J-JL, and YK. Writing of the manuscript: J-HS.

## Conflict of Interest Statement

The authors declare that the research was conducted in the absence of any commercial or financial relationships that could be construed as a potential conflict of interest.

## Abbreviations

GI, gastrointestinal; HP, *Helicobacter pylori*; TTH, tension-type headache; CDW, clinical data warehouse; EMR, electronic medical record; GERD, gastroesophageal reflex disorder; CSG, chronic superficial gastritis; LA class, Los Angeles classification; IBS, irritable bowel syndrome; ANS, autonomic nervous system; NSAIDs, non-steroidal anti-inflammatory drugs.
